# Surgical outcomes of neoadjuvant endocrine treatment in early breast cancer: meta-analysis

**DOI:** 10.1093/bjsopen/zrae100

**Published:** 2024-10-18

**Authors:** Beatrice Brett, Constantinos Savva, Bahar Mirshekar-Syahkal, Martyn Hill, Michael Douek, Ellen Copson, Ramsey Cutress

**Affiliations:** Cancer Sciences, Faculty of Medicine, University of Southampton and University Hospital Southampton, Southampton, UK; Cancer Sciences, Faculty of Medicine, University of Southampton and University Hospital Southampton, Southampton, UK; Cambridge Breast Unit, Cambridge University Hospitals NHS Foundation Trust, Cambridge, UK; Nuffield Department of Surgical Sciences, University of Oxford and John Radcliffe Hospital, Oxford, UK; Nuffield Department of Surgical Sciences, University of Oxford and John Radcliffe Hospital, Oxford, UK; Cancer Sciences, Faculty of Medicine, University of Southampton and University Hospital Southampton, Southampton, UK; Cancer Sciences, Faculty of Medicine, University of Southampton and University Hospital Southampton, Southampton, UK

## Abstract

**Background:**

Neoadjuvant endocrine therapy presents an important downstaging option with lower toxicity than neoadjuvant chemotherapy in oestrogen receptor (ER)-positive early breast cancer. Meta-analysis of the effects of neoadjuvant endocrine therapy on surgical outcomes across randomized clinical trials (RCTs) and cohort studies has not previously been performed.

**Methods:**

A systematic review and meta-analysis was performed to evaluate the effect of neoadjuvant endocrine therapy on surgical outcomes (PROSPERO (international prospective register of systematic reviews, 2020)) compared with surgery followed by adjuvant endocrine therapy. PubMed and EMBASE were searched to identify RCT and cohort studies between 1946 and 27 March 2024. Two independent reviewers manually screened the identified records and extracted the data. Risk of bias was assessed using the Cochrane Collaboration tools and random-effects meta-analysis was done with ReviewManager.

**Results:**

The search identified 2390 articles eligible for screening. The review included 20 studies (12 cohort and 8 RCTs); 19 were included in the meta-analysis with a total of 6382 patients. Overall, neoadjuvant endocrine therapy was associated with a lower mastectomy rate compared with surgery first (risk ratio (RR) 0.53, 95% c.i. 0.44 to 0.64). Subgroup analysis showed similar improvement in the mastectomy rate in the neoadjuvant endocrine therapy group *versus* control group irrespective of study type (RCT: RR 0.58, 95% c.i. 0.50 to 0.66; cohorts: RR 0.48, 95% c.i. 0.33 to 0.70). There was no difference in the mastectomy rate by duration of neoadjuvant endocrine therapy (more than 4 months: RR 0.57, 95% c.i. 0.42 to 0.78; 4 months or less than 4 months: RR 0.52, 95% c.i. 0.43 to 0.64). Most of the studies were characterized by moderate-quality evidence with significant heterogeneity.

**Conclusion:**

Neoadjuvant endocrine therapy is associated with a reduction in mastectomy rate. Given the moderate methodological quality of previous studies, further RCTs are required.

**Registration ID:**

CRD42020209257

## Teaser

Meta-analysis of the comparison of neoadjuvant endocrine therapy followed by surgery to upfront surgery followed by adjuvant endocrine therapy is associated with a reduction in mastectomy rate.

## Introduction

Breast cancer is the most common cancer in the UK, accounting for 15% of all new cancer cases in 2017^1^. Breast cancer-specific survival at 10 years has improved significantly over the last two decades^2^. Approximately 70% of breast cancers are oestrogen receptor (ER)-positive and/or progesterone receptor (PR)-positive, with 85% of these tumours in women over 70 years of age, making endocrine therapy an important therapeutic modality^3,4^.

Upfront surgery is the first-line treatment for breast cancer for the majority of patients followed by adjuvant radiotherapy or chemotherapy, guided by histopathological or molecular features. Data from the National Audit of Breast Cancer in Older Patients (NABCOP) suggest that overall 44% of patients over the age of 50 treated by surgery in England and Wales between 2014 and 2016 had a mastectomy^5^.

Mastectomy has been associated with impaired quality of life that is only partially compensated by postmastectomy breast reconstruction^6^. The use of neoadjuvant chemotherapy is associated with improved rates of breast-conserving surgery (BCS) that are then associated with better quality of life^6,7^. Spring *et al.* showed that neoadjuvant endocrine treatment (NET) is equivalent to neoadjuvant chemotherapy (NAC) in terms of response rates but is associated with a significantly lower risk of adverse events, suggesting that NET should be considered in the neoadjuvant setting in certain groups of patients with early ER-positive breast cancer^3^.

It is unclear however if neoadjuvant therapy more generally improves other surgical outcomes such as surgical morbidity rate in the axilla and/or breast, decreases excision volumes, and reduces positive (involved) margin rates^8^. Currently, NET is recommended in women with early stage I or II Luminal A or B invasive breast cancer whose co-morbidities or tumour stage prevent them from undergoing BCS or receiving NAC^9^. In the UK, the National Institute of Health and Care Excellence (NICE) recommends NET ‘as an option to reduce tumour size if there is no definite indication for chemotherapy’^10^ and in the USA the American Society of Clinical Oncology (ASCO) recommends that in postmenopausal patients with hormone receptor-positive and HER2-negative disease ‘neoadjuvant endocrine therapy with an aromatase inhibitor (AI) may be offered to increase locoregional treatment options’^11^. Despite this, the adoption of NET for ER-positive breast tumours has been much slower than other types of systemic neoadjuvant anticancer treatment^4,12^, although presurgical endocrine therapy was widely used as a ‘bridging’ option during the COVID-19 pandemic^13^.

Both NICE and ASCO guidelines stress the importance of NET to optimize locoregional and surgical options. This has not been evaluated in previous meta-analyses across randomized clinical trials (RCTs) and cohort studies as a primary outcome with a comparator to NET of surgery performed in a group having surgery followed by adjuvant ET, or of the baseline surgical plan compared with the post-NET surgery performed. In addition, there is currently no clear evidence regarding the optimal duration of NET in early breast cancer. The aim of this systematic review and meta-analysis was to evaluate the current evidence and assess whether the use of NET is associated with reduced extent of surgery when compared with upfront surgery, and if so determine if there might be evidence for an optimum duration of NET.

## Methods

### Registration

The systematic review was registered in PROSPERO (international prospective register of systematic reviews, 2020; Registration ID: CRD42020209257), which can be accessed at https://www.crd.york.ac.uk/prospero/.

### Study design

The PICOS (Population, Intervention, Comparison, Outcomes and Study designs) table of records searched in this study is described in *Table 1*. RCTs or cohort studies that investigated mastectomy rate in postmenopausal women with ER-positive early breast cancer who were treated with NET *versus* upfront surgery were included in the meta-analysis.

**Table 1:** PICOS table of outcomes reported in this article

Population	Postmenopausal women with ER-positive breast cancer
Intervention	Neoadjuvant endocrine therapy
Comparison	Population receiving surgery as first-line treatment
Outcomes	Primary outcome: mastectomy rate. Secondary outcomes: optimal duration of NET. Comparison of AI and SERM/SERD. Excision weights and excision specimen volumes. Number of operations/repeat surgery. Positive margin rates. Patient-reported outcomes using a validated scale. Oncological outcomes
Study designs	Randomized clinical trials and cohort studies (comparison and single arm)

PICOS, Population, Intervention, Comparison, Outcomes and Study designs; ER, oestrogen receptor; NET, neoadjuvant endocrine treatment; AI, aromatase inhibitors; SERM, selective oestrogen receptor modulators; SERD, selective oestrogen receptor downregulators.

### Literature criteria

This systematic review was carried out according to the PRISMA statement^14^. A comprehensive systematic literature search was performed by screening MEDLINE (via OVID) from 1946 to March 2024, and Embase Classic + Embase (via OVID) from 1947 to March 2024. *Table S1* shows the complete search strategy for MEDLINE. A similar search algorithm was used for Embase. In addition, reference lists of the eligible studies were hand-searched. Additional records were identified through reference screening of recently published systematic reviews.

### Inclusion and exclusion criteria

Inclusion and exclusion criteria were predefined. Only studies published in the English language were included. The settings for inclusion were patients diagnosed and treated within breast cancer units or centres or secondary care. Studies that included patients with ER-negative breast cancer or metastatic breast cancer, preclinical studies such as murine or cell culture studies, window of opportunity studies where endocrine therapy was given for a short interval before surgery with biological endpoints, studies where primary endocrine therapy was given as the only treatment in patients without intent for surgery and studies that had a sample size of less than 30 patients were excluded.

### Screening process

Following importation of the search results into Endnote, duplicates were removed initially by Endnote and then manually. Two researchers independently screened all articles (B.B. and C.S. from 1946 to October 2020; C.S. and B.M. from October 2020 to March 2024), by title, abstract and full text. Discrepancies in title screening were resolved by abstract screening and discrepancies in abstract or full-text screening were decided by a consensus meeting (C.S., B.B., B.M., E.C., R.I.C.).

### Assessment of methodological quality

Two researchers (B.M. and C.S.) independently assessed the methodological quality of the included studies using the Cochrane Grading of Recommendations Assessment, Development and Evaluation tool for RCTs and observational studies^15^.

### Data extraction

The following data were extracted from the included studies into a table composed of nine main sections: first author’s name, year of publication, sample size per group, study design, type of intervention, comparison group, mastectomy rate, response rate and duration of NET. Two researchers (B.B. and C.S. for articles published until October 2020; C.S. and B.M. for articles published from October 2020 to March 2024) independently performed data extraction and resolved inconsistencies by consensus as above.

### Data synthesis and statistical analysis

The primary outcome of the meta-analysis was comparison of the mastectomy rates between the NET and upfront surgery. The secondary outcomes were optimal duration of NET and the comparison of AIs and selective oestrogen receptor modulators and downregulators (SERM/Ds). RevMan 5.4 (Cochrane Collaboration, UK) was used for the meta-analysis^16^. Data for other secondary outcomes such as patient-reported outcomes, excision weight and volume of surgical specimen as well as oncological outcomes were insufficient and hence, they were not included in the meta-analysis. For each intervention and outcome pair, the summary effect (risk ratio (RR)) and the 95% confidence interval using the random-effects method was utilized.

To assess potential heterogeneity stratified analysis by study design, duration and type of NET was conducted. To evaluate possible heterogeneity between studies, the *I*^2^ statistic, which reflects the proportion of the total variation across studies beyond chance^17^, was applied. A decision threshold was required so after a review of the literature a threshold of 50% for *I*^2^ was selected^18,19^. Consequently, an *I*^2^ value of more than 50% was deemed an indication of high heterogeneity^19^. The 95% prediction intervals were also calculated^20^. To detect potential publication bias, funnel plots were created for the calculated meta-analyses^21^.

## Results

### Characteristics of the included studies

**Fig. 1:**
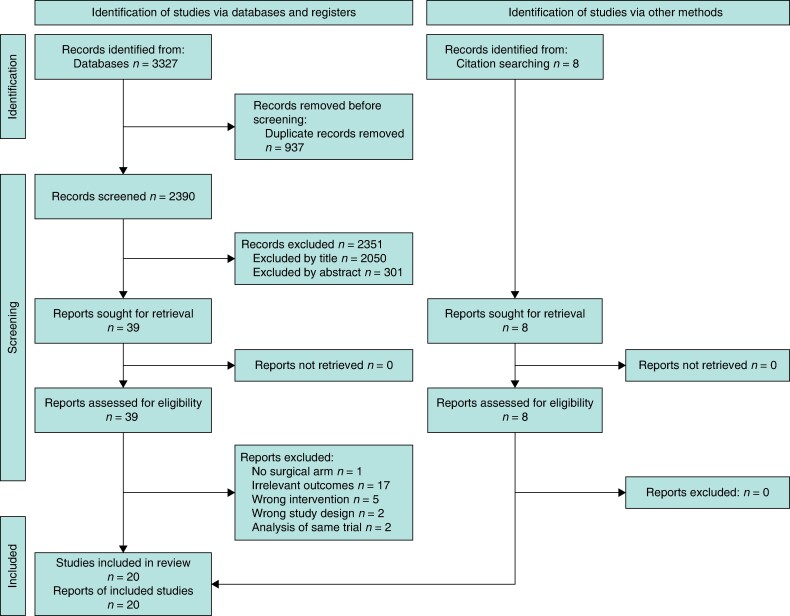
PRISMA flow diagram of included and excluded articles for the systematic review and meta-analysis

The literature search revealed a total of 2390 unique records. After title, abstract and full-text review 20 studies were included in the review^22–41^ (*Fig. 1*). A total of 6382 patients, 3228 in the pre-NET group *versus* 3154 in the post-NET group, that corresponded to 19 studies, were included in the meta-analysis. The study by Chiba *et al*. (remaining study) was included in the systematic review but not in the meta-analysis (except in a sensitivity analysis) because this study differed in the design as it was the only large two-arm study with a surgical control where 74 978 patients received primary surgery and 2294 patients were treated with NET^24^. The study design characteristics of the included studies are shown in *Tables 2* and *3*, where eight single-arm RCTs, 10 single-arm prospective cohorts, one single-arm retrospective cohort and one two-arm retrospective cohort examined mastectomy rates in patients that received NET *versus* upfront surgery. The clinical characteristics of the eligible studies are described in *Table S2*.

**Table 2:** Study design characteristics of the individual studies included in the meta-analysis

Author	Year	Country	NET sample size	Control sample size	Total sample size	Design	Arms	Control	NET
Carpenter *et al*.^22^	2014	UK	146	146	292	Cohort	Single arm	BSD	Letrozole
Cataliotti *et al*.^23^	2006	Multinational	262	314	576	RCT	Single arm	BSD	Tamoxifen or Anastrozole
Chiba *et al*.^24^	2017	USA	2294	74 978	77 272	Cohort	Two arms	Surgical arm	Any NET
Dixon *et al*.^26^	2009	UK	182	182	364	Cohort	Single arm	BSD	Letrozole
Dixon *et al*.^25^	2011	UK	41	61	102	Cohort	Single arm	BSD	Letrozole
Eiermann *et al*.^27^	2001	Multinational	324	324	648	RCT	Single arm	BSD	Letrozole
Ellis *et al*.^28^	2011	USA	352	352	704	RCT	Single arm	BSD	Anastrozole, Letrozole or Exemestane
Fasching *et al*.^29^	2014	Germany	156	156	312	RCT	Single arm	BSD	Letrozole
Fontein *et al*.^30^	2014	Netherlands	102	102	204	Cohort	Single arm	BSD	Exemestane
Hojo *et al*.^31^	2013	Japan	50	52	102	RCT	Single arm	BSD	Exemestane
Hunt *et al*.^32^	2023	USA	509	509	509	Cohort	Single arm	BSD	Anastrozole, Letrozole or Exemestane
Iwata *et al*.^33^	2019	Japan	197	197	394	Cohort	Single arm	BSD	Letrozole
Kantor *et al*.^34^	2020b	USA	94	94	188	Cohort	Single arm	BSD	Not reported
Krainick-Strobel *et al*.^35^	2008	Germany	32	32	64	Cohort	Single arm	BSD	Letrozole
Murphy *et al*.^36^	2021	USA	186	186	186	Cohort	Single arm	BSD	Aromatase inhibitor or tamoxifen ± Goserelin
Olson *et al*.^37^	2009	USA	96	96	192	Cohort	Single arm	BSD	Letrozole
Quenel-Tueux *et al*.^38^	2015	France	108	108	216	RCT	Single arm	BSD	Anastrozole or Fulvestrant
Semiglazov *et al*.^39^	2007	Russia	121	121	242	RCT	Single arm	BSD	Anastrozole or Exemestane
Smith *et al*.^40^	2005	UK and Germany	124	124	248	RCT	Single arm	BSD	Anastrozole or Tamoxifen
Ueno *et al*.^41^	2014	Japan	64	64	128	Cohort	Single arm	BSD	Exemestane

RCT, randomized clinical trial; BSD, baseline surgical decision; NET, neoadjuvant endocrine therapy.

**Table 3:** Total sample sizes of the meta-analysis by study type

Arms	Design	NET sample size	Control sample size	Total
Single arm	Cohort	1657	1677	3334
	RCT	1497	1551	3048
	Total	3154	3228	6382
Two arms	Cohort	2294	74 978	77 272
	RCT	0	0	0
	Total	2294	74 978	77 272
Grand Total		5448	78 206	83 654

NET, neoadjuvant endocrine therapy; RCT, randomized clinical trial.

To validate the results against previous reports, the objective response rates of SERM/Ds *versus* AIs were examined. This analysis revealed that the use of AI is associated with a 14% higher response rate compared with SERM/Ds (RR 1.14, 95% c.i. 1.05 to 1.23) that is comparable with the meta-analysis by Spring *et al.*^3^ (*Fig. S1a*). Nevertheless, the analysis included the study by Quenel-Tueux *et al*.^38^ and did not include the studies by Harper-Wynne *et al.*^42^ and Masuda *et al*.^43^ due to the different primary objectives and criteria of the two meta-analyses. Analysis of the BCS rate by type of NET showed that the use of AI is correlated with a 21% higher BCS rate compared with SERM/Ds (RR 1.21, 95% c.i. 1.09 to 1.34) (*Fig. S1b*).

### Summary effect size and stratified analyses

**Fig. 2:**
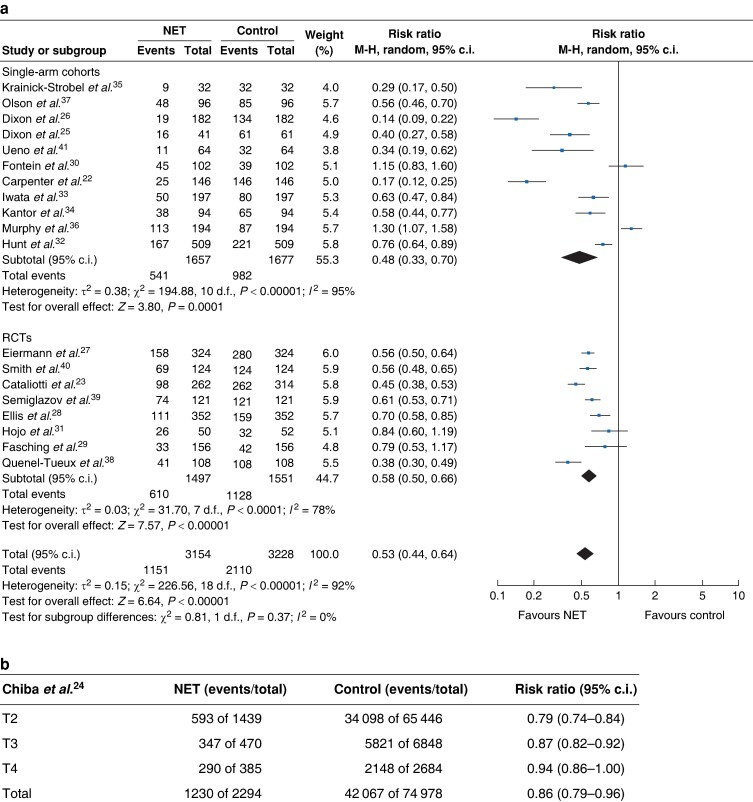
a Forest plot random-effects meta-analysis comparing mastectomy rate at baseline and after neoadjuvant endocrine treatment in patients with early breast cancer stratified by study design. b Mastectomy rate postneoadjuvant endocrine treatment in the Chiba *et al*. cohort (NET *versus* surgical control) stratified by T-stage at diagnosis

After including all studies in the meta-analysis, NET was associated with a 47% reduction in the mastectomy rate compared with the baseline surgical decision (RR 0.53, 95% c.i. 0.44 to 0.64) (*Fig. 2a*). Chiba *et al*. demonstrated that NET was associated with a 14% lower mastectomy rate compared with controls, an effect that was higher in patients with tumours less than 5 cm (RR 0.79, 95% c.i. 0.74 to 0.84) (*Fig. 2b*). To assess the robustness of the data, sensitivity analysis was conducted including the Chiba study that showed similar results (RR 0.55, 95% c.i. 0.45 to 0.66) (*Fig. S2*).

NET, neoadjuvant endocrine treatment.

**Fig. 3:**
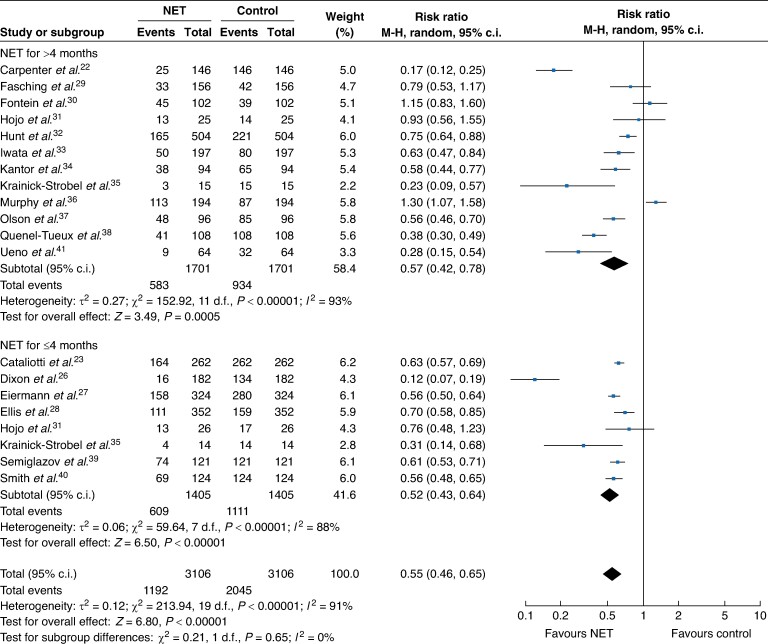
Forest plot random-effects meta-analysis comparing mastectomy rate at baseline and after neoadjuvant endocrine treatment in patients with early breast cancer in all included studies stratified by duration of endocrine therapy NET, neoadjuvant endocrine treatment.

Stratified analysis by type of study showed similar effects between RCTs and cohort studies, with the single-arm cohort studies having wider confidence intervals attributed to their smaller sample size. Specifically, in the single-arm cohort studies there was a 52% reduction in the mastectomy rate (RR 0.48, 95% c.i. 0.33 to 0.70), whilst in the RCTs the mastectomy rate was decreased by 42% (0.58, 95% c.i. 0.50 to 0.66) compared with the controls (*Fig. 2a*). Stratified analysis comparing mastectomy rate at baseline and after NET by duration of endocrine therapy showed no statistically significant differences between more than 4 months *versus* 4 months or less of NET (more than 4 months: RR 0.57, 95% c.i. 0.42 to 0.78; equal to or less than 4 months: RR 0.52, 95% c.i. 0.43 to 0.64) (*Fig. 3*).

### Between-study heterogeneity and publication bias

The association between NET and mastectomy rate showed a high level of heterogeneity that was statistically significant across all studies and after they were stratified by study design and duration of NET (*Figs. 2*, *3*).The heterogeneity can be explained by methodological differences such as differences in inclusion criteria (especially relating to operability), the sample size and eligibility for mastectomy. The study design also contributed to the heterogeneity as there was a difference in the risk ratio for the rate of mastectomy among the different study types (*Fig. 2a,b*). Furthermore, mastectomy rate was not the primary outcome of many of these studies, which might also account for methodological differences among these different studies. Regarding clinical and biological heterogeneity, the included studies were comparable in relation to the mean age of the participants, menopausal status and tumour size. Nevertheless, tumour histological grade and HER2 profile were not reported in seven and nine studies respectively, and nine studies included patients with HER2-positive tumours (*Table S2*).

Visual inspection of the funnel plot of the 20 studies included in the meta-analysis showed an asymmetry of association between NET and mastectomy rates for the RCTs and a symmetry for the cohort studies (*Fig. S3a*). When these studies were stratified by HER2 status (known *versus* unknown), there was an asymmetry between RCTs and single-arm cohorts in the group where the HER2 profile was not reported (*Fig. S3b*,*c*). Most of the modern studies (2011 to 2024) reported HER2 status whereas HER2 expression was not reported in the majority of older studies (2006 to 2014).

### Methodological quality of the studies

Grading of the level of evidence of both cohort studies and RCTs using the Cochrane GRADE (Grading of Recommendations Assessment, Development and Evaluation) tool showed that these studies were characterized by moderate methodological quality overall (*Fig. S4*). Selective reporting, random error and measurement of prognostic factors or confounders were categorized as high risk in two^25,30^, three^30,34,35^ and four^25,26,30,35^ cohorts respectively. In RCTs, selection, performance, detection and reporting biases were categorized as high risk in three^29,38,39^, four^29,31,38,39^, three^31,38,39^, and one^29^ study/ies respectively.

## Discussion

NET is recognized as a means of increasing surgical options in ER-positive tumours in postmenopausal women, however, the evidence base for this is limited. The ‘Getting It Right First Time (GIRFT)’ report for breast surgery recommends that multidisciplinary teams should support BCS independently of age and consider primary systemic treatments to facilitate BCS when clinically indicated^44^. Although the effect of NET on the type of surgery has been reported as a secondary outcome in some studies, no sufficiently powered randomized trial data have been published.

Overall, the results of the meta-analysis presented here of 6382 patients receiving NET show that NET is associated with lower mastectomy rates when compared with upfront surgery. This finding held true in both the RCT and single-arm cohort subgroup analyses. Furthermore, the effect was present for both durations of NET that were studied (up to 4 months and greater than 4 months). AIs were associated with a higher BCS rate than SERMS/SERDs, which is in agreement with a previous meta-analysis examining the effect of NET on tumour response and type of surgery^3^, although the current meta-analysis includes a more recent paper published by Quenel-Tueux *et al.*^38^. The main difference between this meta-analysis and the one published by Spring *et al.*^3^ is that the type of surgery planned at baseline has been used as a proxy for the standard of care of surgery first and this has been compared with the type of surgery performed after NET. By contrast, Spring *et al.*^3^ compared BCS rates between different types of NET, or NET *versus* either neo-adjuvant chemotherapy (NACT) or dual therapy. The present study, therefore, adds further information as it is representative of the question faced in the surgical setting to what might be the likelihood of downstaging to breast conservation with NET compared with if patients proceed directly to primary surgery.

The study protocol was preregistered and identified all relevant studies from the published literature. There is a possibility that some studies were excluded because they were not in the English language.

There was significant heterogeneity between the studies. This is likely to be due to both the characteristics of the included patients and their tumours and the type and duration of NET, particularly as some studies gave a range of durations or altered the duration based on the response. In addition, there are well documented issues with adherence to endocrine therapy^45^, although this is more of a problem in the adjuvant setting, when endocrine therapy is prescribed for several years. The definition of ER-positive was not uniform amongst the studies, and not all studies included HER2 status, factors that are known to affect response to NET. In addition, more recent studies that reported HER2 status would have more likely given neoadjuvant anti-HER2 targeted therapy compared with studies that did not report the status of the HER2 receptor.

The findings of this meta-analysis support both the current NICE and ASCO guidelines that recommend considering or offering the use of NET to downstage ER-positive and HER2-negative tumours and increase local treatment options in postmenopausal women if there is no definite indication for chemotherapy^10,11^. These recommendations have mainly been derived from studies that showed increased BCS with NACT compared with adjuvant chemotherapy^46–48^. In the NICE guideline (2023), NET is presented as an option for postmenopausal women where chemotherapy is not definitely indicated based on the preoperative findings^10^. This is despite the suggestion from Spring *et al.*^3^ that the two are likely to be equivalent, as well as the more superior side effect profile and ease of administration of NET that is highlighted in both guidelines. Reasons for favouring neoadjuvant chemotherapy where chemotherapy is indicated in postmenopausal women include the longer duration of treatment required with NET and the lower complete pathological response rate. The evidence for NET in premenopausal women is less clear, and the neoadjuvant preference recommendation for premenopausal women is for NACT if chemotherapy is indicated. The latter is supported by a trial by Alba *et al.* that reported higher response rates for NACT compared with NET in premenopausal women, whereas this association was not observed in postmenopausal patients^49^. All the studies included in the present meta-analysis apart from Kantor *et al.*^34^ were of postmenopausal patients.

A survey in patients older than 70 years of age showed that 20% of those who underwent mastectomy would consider BCS had it been an option, and 40% would be willing to receive NET to downstage their breast cancer if it facilitated BCS^50^. Hence, the intention was to analyse additional outcomes associated with NET, such as health-related quality of life (HRQoL) measures, oncological and surgical outcomes, however, there was little available information on outcomes other than the response to NET and the type of surgery. Taira *et al.* studied HRQoL in a Japanese population as part of the New primary Endocrine therapy Origination Study (NEOS) trial and reported no difference in global HRQoL scores^51^. They found increased hot flushes and reduced social and family wellbeing scores as well as improved emotional wellbeing over the 16-week time interval studied; however, they did not compare this to upfront surgery. Follow-up of the JFMC34–0601 neoadjuvant endocrine study^41^ found an independent association between clinical response (classed as partial response, stable or progressive disease because there were no instances of complete clinical response in the trial) and disease-free survival, distant disease-free survival and overall survival, with progressive disease in particular being associated with a poor prognosis^52^. Oncological outcomes were also evaluated by Kantor *et al*., where there was no difference in 5-year overall survival between patients with minimal residual nodal disease and those with node-negative disease after NET^53^. These findings reflected the outcome of patients who received upfront surgery when compared with a matched cohort of patients that received surgery first^53^, supporting the oncological safety of NET. Multivariable analysis showed that post-NET high-risk histopathological features such as high histological tumour grade, PR negativity, tumour size of greater than 5 cm and residual macrometastatic nodal disease were independently associated with shorter survival that improved significantly with the use of adjuvant chemotherapy^53^. This suggests that the adjuvant treatment of patients without adverse histopathological features post-NET may possibly be de-escalated.

The main limitation of the meta-analysis reported here is that most of the studies did not include details of how the decision for surgery was made and by whom. Most studies did not set out to measure mastectomy rates *per se* and focused on tumour response. Indeed, there is no standardized method for deciding the type of surgery performed as this is ultimately a joint decision between the clinician and the patient, hence the risk of bias was deemed unclear in papers that did not explicitly describe how the surgical decision was made. Moreover, for all studies included in the meta-analysis, except the study by Chiba *et al.*^24^, the baseline surgical decision before starting NET was used as the comparator to final surgery. This may have underestimated the number of mastectomies that would have been required at baseline as some patients may have been converted from BCS to mastectomy due to involved margins on histology. Furthermore, the studies had different eligibility criteria regarding the extent of disease at baseline, with some including those who were inoperable who had NET with a view to downstaging to operability^23,25–28,37^, and it can be envisaged that these patients may require a more convincing response to NET in order to be deemed eligible for BCS. This is supported by the Chiba *et al.* study, which showed reduced effect of NET on mastectomy rates with increasing T stage^24^.

The published literature suggests NET is associated with a reduction in the rate of mastectomy by around half compared with baseline surgical decision. There was, however, significant heterogeneity in data used for this meta-analysis. In order to guide clinical practice, future work should compare NET with upfront surgery, measuring type of surgery as a primary outcome.

The findings in this systematic review and meta-analysis should be interpreted with caution as the studies included are characterized by methodological and biological heterogeneity and moderate methodological quality. An RCT is required to evaluate the role of NET in improving surgical outcomes. The present analysis indicates that the use of AI is superior to SERM/Ds in the NET setting, however, the optimal duration of NET is not clear.

## Supplementary Material

zrae100_Supplementary_Data

## Data Availability

The data that support the findings of this study are derived from public domain sources and are openly available within MEDLINE and Embase.
