# Supplementary material for: Surgical outcomes of neoadjuvant endocrine treatment in early breast cancer: meta-analysis
Source: BJS Open. 2024 Oct 18;8(5):zrae100. doi: 10.1093/bjsopen/zrae100 (PMC11488384; doi:10.1093/bjsopen/zrae100)
Supplement: zrae100_Supplementary_Data [file zrae100_supplementary_data.zip › Supplementary Material_Tables 26.5.2024.docx]

**Surgical outcomes of neoadjuvant endocrine treatment in early breast cancer: A Systematic Review and Meta-analysis**

Authors: Brett Beatrice^#1^, Savva Constantinos^#^*^1^, Bahar Mirshekar-Syahkal^2^, Hill Martyn^3^, Douek, Michael^3^, Copson Ellen^1^ and Cutress Ramsey*^1^

*Corresponding Authors

#Contributed equally and so should be considered joint first authors

Author Affiliations:

^1^ Cancer Sciences, Faculty of Medicine, University of Southampton and University Hospital Southampton, Southampton SO16 6YD, UK.

^2^ Cambridge Breast Unit, Cambridge University Hospitals NHS Foundation Trust, Cambridge, United Kingdom, CB2 0QQ, UK.

^3^ Nuffield Department of Surgical Sciences, University of Oxford and John Radcliffe Hospital, Headington, Oxford, OX3 9DU, UK.

*Corresponding authors:

Dr Constantinos Savva and Professor Ramsey Cutress. Somers Cancer Sciences Building, Southampton General Hospital, Tremona Road, Southampton SO16 6YD, UK.

E-mail: [c.savva@soton.ac.uk](mailto:c.savva@soton.ac.uk) and [R.I.Cutress@soton.ac.uk](mailto:R.I.Cutress@soton.ac.uk)

**Supplementary Materials - Index**

| **Supplementary Tables** |  |
| --- | --- |
| Supplementary table 1 | *page 2* |
| Supplementary table 2 | *page 6* |
|  |  |
|  |  |
|  |  |

**Supplementary Tables**

***Supplementary table 1.*** *Search strategy.*

|  | Search terms |
| --- | --- |
| 1 | Breast Neoplasms/ |
| 2 | ((mammary or breast) adj3 (cancer* or neoplas* or carcinoma* or malignan*)).mp. [mp=title, abstract, heading word, drug trade name, original title, device manufacturer, drug manufacturer, device trade name, keyword, floating subheading word, candidate term word] |
| 3 | ((surg* or cosmetic or aesthetic) adj3 outcome*).mp. [mp=title, abstract, heading word, drug trade name, original title, device manufacturer, drug manufacturer, device trade name, keyword, floating subheading word, candidate term word] |
| 4 | (re-excision or reexcision).mp. [mp=title, abstract, heading word, drug trade name, original title, device manufacturer, drug manufacturer, device trade name, keyword, floating subheading word, candidate term word] |
| 5 | ((breast or mammary) adj3 reconstruction).mp. [mp=title, abstract, heading word, drug trade name, original title, device manufacturer, drug manufacturer, device trade name, keyword, floating subheading word, candidate term word] |
| 6 | oncoplastic.mp. [mp=title, abstract, heading word, drug trade name, original title, device manufacturer, drug manufacturer, device trade name, keyword, floating subheading word, candidate term word] |
| 7 | ((excision or specimen) adj3 (volume or weight)).mp. [mp=title, abstract, heading word, drug trade name, original title, device manufacturer, drug manufacturer, device trade name, keyword, floating subheading word, candidate term word] |
| 8 | margin.mp. [mp=title, abstract, heading word, drug trade name, original title, device manufacturer, drug manufacturer, device trade name, keyword, floating subheading word, candidate term word] |
| 9 | margins.mp. [mp=title, abstract, heading word, drug trade name, original title, device manufacturer, drug manufacturer, device trade name, keyword, floating subheading word, candidate term word] |
| 10 | PROM.mp. [mp=title, abstract, heading word, drug trade name, original title, device manufacturer, drug manufacturer, device trade name, keyword, floating subheading word, candidate term word] |
| 11 | PROMS.mp. [mp=title, abstract, heading word, drug trade name, original title, device manufacturer, drug manufacturer, device trade name, keyword, floating subheading word, candidate term word] |
| 12 | patient reported outcome*.mp. [mp=title, abstract, heading word, drug trade name, original title, device manufacturer, drug manufacturer, device trade name, keyword, floating subheading word, candidate term word] |
| 13 | HRQOL.mp. [mp=title, abstract, heading word, drug trade name, original title, device manufacturer, drug manufacturer, device trade name, keyword, floating subheading word, candidate term word] |
| 14 | health related quality of life.mp. [mp=title, abstract, heading word, drug trade name, original title, device manufacturer, drug manufacturer, device trade name, keyword, floating subheading word, candidate term word] |
| 15 | QLACS.mp. [mp=title, abstract, heading word, drug trade name, original title, device manufacturer, drug manufacturer, device trade name, keyword, floating subheading word, candidate term word] |
| 16 | quality of life in adult cancer survivors.mp. [mp=title, abstract, heading word, drug trade name, original title, device manufacturer, drug manufacturer, device trade name, keyword, floating subheading word, candidate term word] |
| 17 | QLI-CV.mp. [mp=title, abstract, heading word, drug trade name, original title, device manufacturer, drug manufacturer, device trade name, keyword, floating subheading word, candidate term word] |
| 18 | quality of life-cancer version.mp. [mp=title, abstract, heading word, drug trade name, original title, device manufacturer, drug manufacturer, device trade name, keyword, floating subheading word, candidate term word] |
| 19 | QOL-CS.mp. [mp=title, abstract, heading word, drug trade name, original title, device manufacturer, drug manufacturer, device trade name, keyword, floating subheading word, candidate term word] |
| 20 | quality of life-cancer survivors.mp. [mp=title, abstract, heading word, drug trade name, original title, device manufacturer, drug manufacturer, device trade name, keyword, floating subheading word, candidate term word] |
| 21 | survival.mp. [mp=title, abstract, heading word, drug trade name, original title, device manufacturer, drug manufacturer, device trade name, keyword, floating subheading word, candidate term word] |
| 22 | disease free survival.mp. [mp=title, abstract, heading word, drug trade name, original title, device manufacturer, drug manufacturer, device trade name, keyword, floating subheading word, candidate term word] |
| 23 | overall survival.mp. [mp=title, abstract, heading word, drug trade name, original title, device manufacturer, drug manufacturer, device trade name, keyword, floating subheading word, candidate term word] |
| 24 | recurrence rate*.mp. [mp=title, abstract, heading word, drug trade name, original title, device manufacturer, drug manufacturer, device trade name, keyword, floating subheading word, candidate term word] |
| 25 | recurrence*.mp. [mp=title, abstract, heading word, drug trade name, original title, device manufacturer, drug manufacturer, device trade name, keyword, floating subheading word, candidate term word] |
| 26 | QOL.mp. [mp=title, abstract, heading word, drug trade name, original title, device manufacturer, drug manufacturer, device trade name, keyword, floating subheading word, candidate term word] |
| 27 | quality of life.mp. [mp=title, abstract, heading word, drug trade name, original title, device manufacturer, drug manufacturer, device trade name, keyword, floating subheading word, candidate term word] |
| 28 | (hospital anxiety and depression scale).mp. [mp=title, abstract, heading word, drug trade name, original title, device manufacturer, drug manufacturer, device trade name, keyword, floating subheading word, candidate term word] |
| 29 | HADS.mp. [mp=title, abstract, heading word, drug trade name, original title, device manufacturer, drug manufacturer, device trade name, keyword, floating subheading word, candidate term word] |
| 30 | CARES.mp. [mp=title, abstract, heading word, drug trade name, original title, device manufacturer, drug manufacturer, device trade name, keyword, floating subheading word, candidate term word] |
| 31 | functional living index for cancer.mp. [mp=title, abstract, heading word, drug trade name, original title, device manufacturer, drug manufacturer, device trade name, keyword, floating subheading word, candidate term word] |
| 32 | FLIC.mp. [mp=title, abstract, heading word, drug trade name, original title, device manufacturer, drug manufacturer, device trade name, keyword, floating subheading word, candidate term word] |
| 33 | (European organisation for research and treatment of cancer quality of life questionnaire c-30).mp. [mp=title, abstract, heading word, drug trade name, original title, device manufacturer, drug manufacturer, device trade name, keyword, floating subheading word, candidate term word] |
| 34 | EORTC QLQ-C30*.mp. [mp=title, abstract, heading word, drug trade name, original title, device manufacturer, drug manufacturer, device trade name, keyword, floating subheading word, candidate term word] |
| 35 | functional assessment of cancer therapy.mp. [mp=title, abstract, heading word, drug trade name, original title, device manufacturer, drug manufacturer, device trade name, keyword, floating subheading word, candidate term word] |
| 36 | profile of mood states.mp. [mp=title, abstract, heading word, drug trade name, original title, device manufacturer, drug manufacturer, device trade name, keyword, floating subheading word, candidate term word] |
| 37 | POMS.mp. [mp=title, abstract, heading word, drug trade name, original title, device manufacturer, drug manufacturer, device trade name, keyword, floating subheading word, candidate term word] |
| 38 | medical outcomes study short form 36.mp. [mp=title, abstract, heading word, drug trade name, original title, device manufacturer, drug manufacturer, device trade name, keyword, floating subheading word, candidate term word] |
| 39 | MOS-SF-36.mp. [mp=title, abstract, heading word, drug trade name, original title, device manufacturer, drug manufacturer, device trade name, keyword, floating subheading word, candidate term word] |
| 40 | symptom distress scale.mp. [mp=title, abstract, heading word, drug trade name, original title, device manufacturer, drug manufacturer, device trade name, keyword, floating subheading word, candidate term word] |
| 41 | SDS.mp. [mp=title, abstract, heading word, drug trade name, original title, device manufacturer, drug manufacturer, device trade name, keyword, floating subheading word, candidate term word] |
| 42 | Rotterdam symptom checklist.mp. [mp=title, abstract, heading word, drug trade name, original title, device manufacturer, drug manufacturer, device trade name, keyword, floating subheading word, candidate term word] |
| 43 | RSCL.mp. [mp=title, abstract, heading word, drug trade name, original title, device manufacturer, drug manufacturer, device trade name, keyword, floating subheading word, candidate term word] |
| 44 | state trait anxiety index.mp. [mp=title, abstract, heading word, drug trade name, original title, device manufacturer, drug manufacturer, device trade name, keyword, floating subheading word, candidate term word] |
| 45 | STAI.mp. [mp=title, abstract, heading word, drug trade name, original title, device manufacturer, drug manufacturer, device trade name, keyword, floating subheading word, candidate term word] |
| 46 | breast cancer specific quality of life questionnaire.mp. [mp=title, abstract, heading word, drug trade name, original title, device manufacturer, drug manufacturer, device trade name, keyword, floating subheading word, candidate term word] |
| 47 | QLQ-BR23.mp. [mp=title, abstract, heading word, drug trade name, original title, device manufacturer, drug manufacturer, device trade name, keyword, floating subheading word, candidate term word] |
| 48 | breast-Q.mp. [mp=title, abstract, heading word, drug trade name, original title, device manufacturer, drug manufacturer, device trade name, keyword, floating subheading word, candidate term word] |
| 49 | body image scale.mp. [mp=title, abstract, heading word, drug trade name, original title, device manufacturer, drug manufacturer, device trade name, keyword, floating subheading word, candidate term word] |
| 50 | FACT-B*.mp. [mp=title, abstract, heading word, drug trade name, original title, device manufacturer, drug manufacturer, device trade name, keyword, floating subheading word, candidate term word] |
| 51 | BIS.mp. [mp=title, abstract, heading word, drug trade name, original title, device manufacturer, drug manufacturer, device trade name, keyword, floating subheading word, candidate term word] |
| 52 | 1 or 2 |
| 53 | 3 or 4 or 5 or 6 or 7 or 8 or 9 or 10 or 11 or 12 or 13 or 14 or 15 or 16 or 17 or 18 or 19 or 20 or 21 or 22 or 23 or 24 or 25 or 26 or 27 or 28 or 29 or 30 or 31 or 32 or 33 or 34 or 35 or 36 or 37 or 38 or 39 or 40 or 41 or 42 or 43 or 44 or 45 or 46 or 47 or 48 or 49 or 50 |
| 54 | Tamoxifen/ or Aromatase Inhibitors/ |
| 55 | hormon*.mp. [mp=title, abstract, heading word, drug trade name, original title, device manufacturer, drug manufacturer, device trade name, keyword, floating subheading word, candidate term word] |
| 56 | letrozole.mp. [mp=title, abstract, heading word, drug trade name, original title, device manufacturer, drug manufacturer, device trade name, keyword, floating subheading word, candidate term word] |
| 57 | anastrazole.mp. [mp=title, abstract, heading word, drug trade name, original title, device manufacturer, drug manufacturer, device trade name, keyword, floating subheading word, candidate term word] |
| 58 | exemestane.mp. [mp=title, abstract, heading word, drug trade name, original title, device manufacturer, drug manufacturer, device trade name, keyword, floating subheading word, candidate term word] |
| 59 | tamoxifen.mp. [mp=title, abstract, heading word, drug trade name, original title, device manufacturer, drug manufacturer, device trade name, keyword, floating subheading word, candidate term word] |
| 60 | aromatase inhibitor*.mp. [mp=title, abstract, heading word, drug trade name, original title, device manufacturer, drug manufacturer, device trade name, keyword, floating subheading word, candidate term word] |
| 61 | endocrine therap*.mp. [mp=title, abstract, heading word, drug trade name, original title, device manufacturer, drug manufacturer, device trade name, keyword, floating subheading word, candidate term word] |
| 62 | endocrine treat*.mp. [mp=title, abstract, heading word, drug trade name, original title, device manufacturer, drug manufacturer, device trade name, keyword, floating subheading word, candidate term word] |
| 63 | 54 or 55 or 56 or 57 or 58 or 59 or 60 or 61 or 62 |
| 64 | Neoadjuvant Therapy/ |
| 65 | (neoadjuvant or neo adjuvant or neo-adjuvant).mp. [mp=title, abstract, heading word, drug trade name, original title, device manufacturer, drug manufacturer, device trade name, keyword, floating subheading word, candidate term word] |
| 66 | (preoperative or pre operative or pre-operative).mp. [mp=title, abstract, heading word, drug trade name, original title, device manufacturer, drug manufacturer, device trade name, keyword, floating subheading word, candidate term word] |
| 67 | (presurg* or pre-surg* or pre surg*).mp. [mp=title, abstract, heading word, drug trade name, original title, device manufacturer, drug manufacturer, device trade name, keyword, floating subheading word, candidate term word] |
| 68 | 64 or 65 or 66 or 67 |
| 69 | Neoplasm Metastasis/ |
| 70 | (metastasis or metastases or metastatic).mp. [mp=title, abstract, heading word, drug trade name, original title, device manufacturer, drug manufacturer, device trade name, keyword, floating subheading word, candidate term word] |
| 71 | (stage 4 or stage four).mp. [mp=title, abstract, heading word, drug trade name, original title, device manufacturer, drug manufacturer, device trade name, keyword, floating subheading word, candidate term word] |
| 72 | 69 or 70 or 71 |
| 73 | 52 and 53 and 63 and 68 |
| 74 | 73 not 72 |

***Supplementary table 2.*** *Baseline clinical characteristics of all included studies.*

| Author | Age (years)  Mean (SD) | Post-menopausal  n/N | T >20 mm n/N | Grade 3  n/N | HER2+  n/N |
| --- | --- | --- | --- | --- | --- |
| Carpenter et al. (22) | 73.5 (9.7) | 146/146 | 138/139 | 16/139 | NR |
| Cataliotti et al. (23) | 67 | 576/576 | NR | NR | NR |
| Chiba et al. (24) | 66.8 (11.1) | NR | 77,272/77,272 | 21,451/77,272 | NR |
| Dixon et al. (26) | 76.5 (55-95) | 182/182 | 172/182 | NR | NR |
| Dixon et al. (25) | 76.2 (55-91) | 63/63 | 63/63 | 12/63 | 8/63 |
| Eiermann et al. (27) | 68* | 324/324 | 324/324 | NR | NR |
| Ellis et al. (28) | NR | 374/374 | 374/374 | 56/374 | 33/374 |
| Fasching et al. (29) | 70.8 (8.7) | 131/131 | 117/131 | 17/131 | NR |
| Fontein et al. (30) | 72* (53-88) | 72/72 | 59/72¥ | 7/72 | 9/72 |
| Hojo et al. (31) | 65* | 52/52 | 52/52 | NR | 4/52 |
| Hunt et al. (32) | 66* | 509/509 | 509/509 | 66/509 | 29/509 |
| Iwata et al. (33) | 63* (49-75) | 295/295 | 251/295 | 61/295 | 9/252 |
| Kantor et al. (34) | 64.0 (13.0) | NR | 70/94 | 13/94 | 0/94 |
| Krainick-Strobel et al. (35) | 68.4 (7.2) | 32/32 | 32/32 | 2/32 | NR |
| Murphy et al. (36) | 66* | NR | 150/194 | 17/194 | 2/194 |
| Olson et al. (37) | 67 (48-89) | 106/106 | 106/106 | 27/106 | 8/106 |
| Quenel-Tueux et al. (38) | 70* | 120/120 | 120/120 | 8/120 | 7/120 |
| Semiglazov et al. (39) | 68* | 121/121 | 121/121 | NR | NR |
| Smith et al. (40) | 73.2* (52-86) | 109/109 | 24* (10-110) | NR | NR |
| Ueno et al. (41) | NR | 107/107 | 107/107 | NR | 0/107 |
| **Abbreviations:** *median; NR, not reported; ¥>30 mm. | | | | | |
