# Supplementary material for: Surgical outcomes of neoadjuvant endocrine treatment in early breast cancer: meta-analysis
Source: BJS Open. 2024 Oct 18;8(5):zrae100. doi: 10.1093/bjsopen/zrae100 (PMC11488384; doi:10.1093/bjsopen/zrae100)
Supplement: zrae100_Supplementary_Data [file zrae100_supplementary_data.zip › Supplementary_Material.docx]

**Surgical outcomes of neoadjuvant endocrine treatment in early breast cancer: meta-analysis**

Authors: Brett Beatrice^#1^, Savva Constantinos^#^*^1^, Bahar Mirshekar-Syahkal^2^, Hill Martyn^3^, Douek, Michael^3^, Copson Ellen^1^ and Cutress Ramsey*^1^

*Corresponding Authors

#Contributed equally and so should be considered joint first authors

Author Affiliations:

^1^ Cancer Sciences, Faculty of Medicine, University of Southampton and University Hospital Southampton, Southampton SO16 6YD, UK.

^2^ Cambridge Breast Unit, Cambridge University Hospitals NHS Foundation Trust, Cambridge, United Kingdom, CB2 0QQ, UK.

^3^ Nuffield Department of Surgical Sciences, University of Oxford and John Radcliffe Hospital, Headington, Oxford, OX3 9DU, UK.

*Corresponding authors:

Dr Constantinos Savva and Professor Ramsey Cutress. Somers Cancer Sciences Building, Southampton General Hospital, Tremona Road, Southampton SO16 6YD, UK.

E-mail: [c.savva@soton.ac.uk](mailto:c.savva@soton.ac.uk) and [R.I.Cutress@soton.ac.uk](mailto:R.I.Cutress@soton.ac.uk)

**Supplementary Materials - Index**

|  |  |
| --- | --- |
| **Supplementary Figures**  Supplementary Figure 1  Supplementary Figure 2  Supplementary Figure 3  Supplementary Figure 4 | *page 7*  *page 7*  *page 8*  *page 9* |

**Supplementary Figure 1**

Forest plot random effects meta-analysis comparing A, objective response and B, breast conservation rates between selective oestrogen modulator/degrader (tamoxifen and fulvestrant) versus aromatase inhibitors (letrozole and anastrozole) groups in patients with early breast cancer that received neoadjuvant endocrine treatment.

**
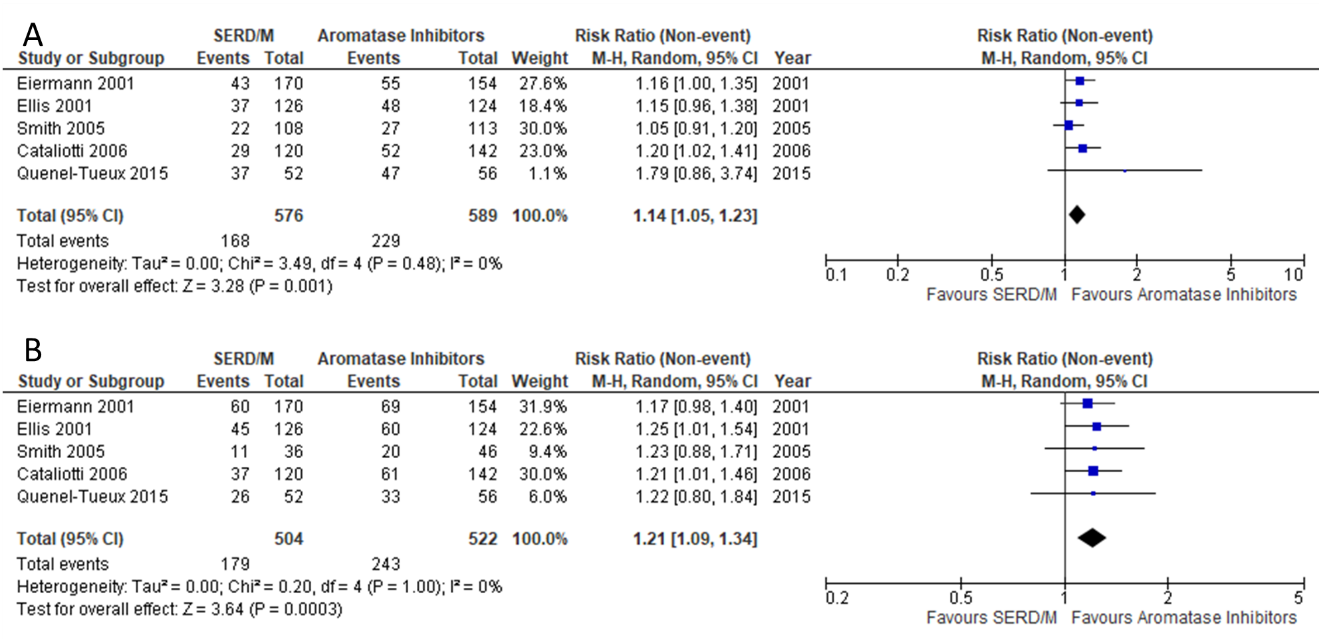
**

**Supplementary Figure 2**

Forest plot random effects meta-analysis comparing mastectomy rate at baseline and after neoadjuvant endocrine treatment in patients with early breast cancer after adding study by Chiba et al.

**
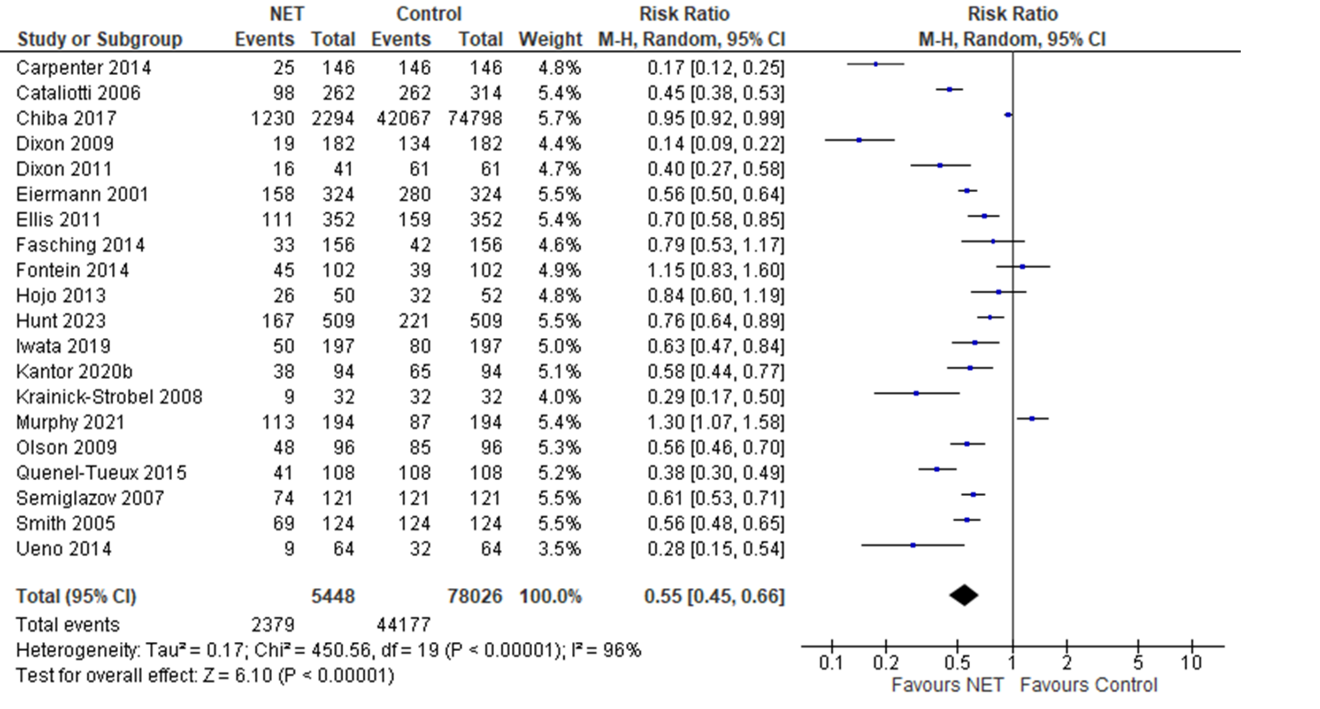
**

**Supplementary Figure 3**

Funnel plots of A, all included studies, B, studies that reported HER2 profile and C, studies that did not report HER2 profile, comparing mastectomy rate at baseline versus neoadjuvant endocrine treatment by study design.

**
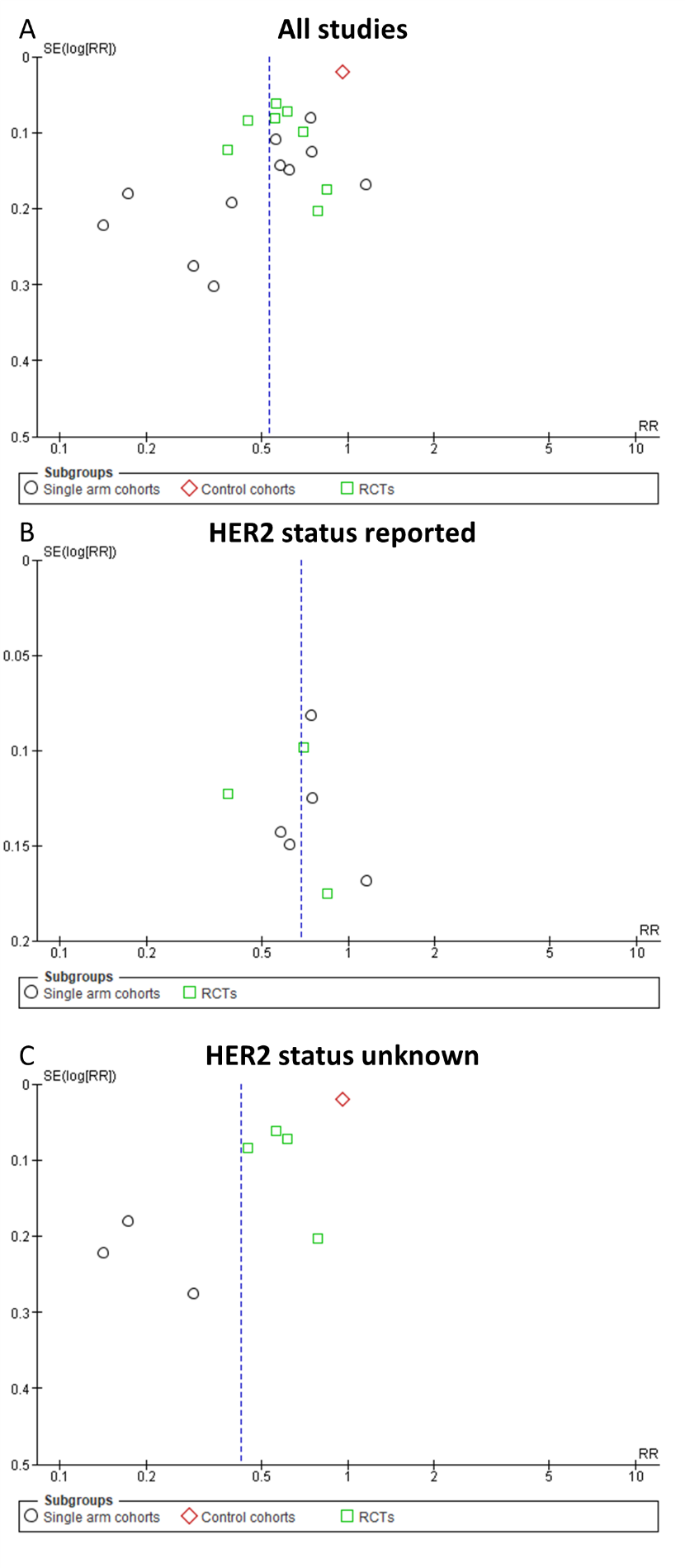
**

**Supplementary Figure 4**

Risk of bias presented as percentages across all included cohorts (A) and randomised controlled trials (B).

**
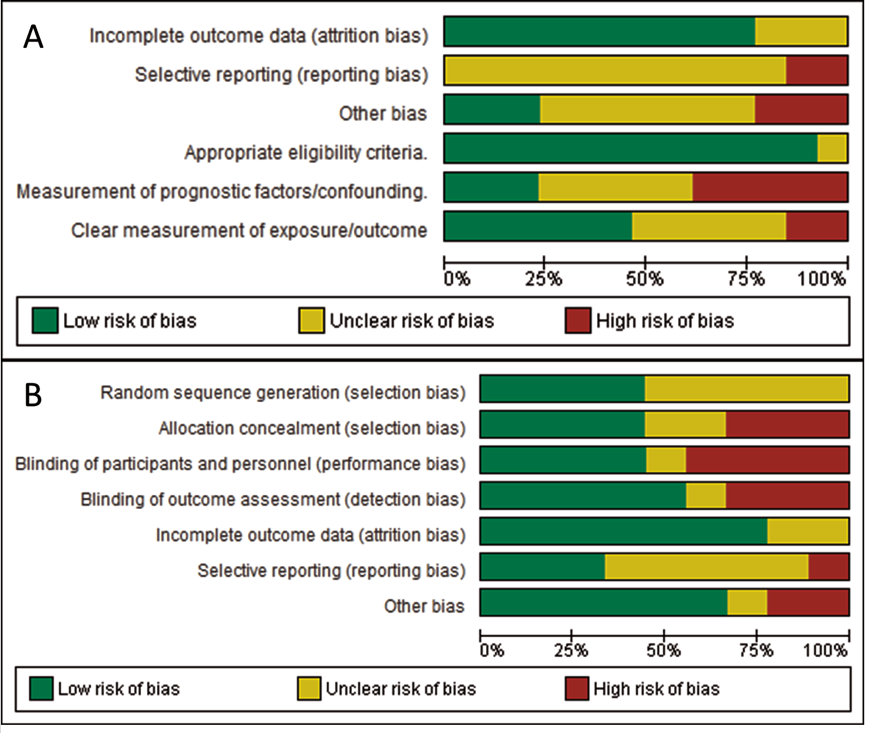
**
